# Treatment with Activated Protein C (aPC) Is Protective during the Development of Myocardial Fibrosis: An Angiotensin II Infusion Model in Mice

**DOI:** 10.1371/journal.pone.0045663

**Published:** 2012-09-19

**Authors:** Mryanda J. Sopel, Nicole L. Rosin, Alec G. Falkenham, Michael Bezuhly, Charles T. Esmon, Timothy D. G. Lee, Robert S. Liwski, Jean-Francois Légaré

**Affiliations:** 1 Department of Pathology, Dalhousie University, Halifax, Nova Scotia, Canada; 2 Department of Surgery, Dalhousie University, Halifax, Nova Scotia, Canada; 3 Department of Microbiology and Immunology, Dalhousie University, Halifax, Nova Scotia, Canada; 4 Howard Hughes Medical Institute and Cardiovascular Biology Research Program, Oklahoma City, Oklahoma, United States of America; 5 Oklahoma Medical Research Foundation, Oklahoma City, Oklahoma, United States of America; University of Western Ontario, Canada

## Abstract

**Aims:**

Myocardial fibrosis contributes to the development of heart failure. Activated Protein C (aPC) is a circulating anticoagulant with anti-inflammatory and cytoprotective properties. Using a model of myocardial fibrosis second to Angiotensin II (AngII) infusion, we investigated the novel therapeutic function aPC in the development of fibrosis.

**Methods and Results:**

C57Bl/6 and Tie2-EPCR mice were infused with AngII (2.0 µg/kg/min), AngII and aPC (0.4 µg/kg/min) or saline for 3d. Hearts were harvested and processed for analysis or used for cellular isolation. Basic histology and collagen deposition were assessed using histologic stains. Transcript levels of molecular mediators were analyzed by quantitative RT-PCR. Mice infused with AngII exhibited multifocal areas of myocardial cellular infiltration associated with significant collagen deposition compared to saline control animals (*p*<0.01). AngII-aPC infusion inhibited this cellular infiltration and the corresponding collagen deposition. AngII-aPC infusion also inhibited significant expression of the pro-fibrotic cytokines TGF-β1, CTGF and PDGF found in AngII only infused animals (p<0.05). aPC signals through its receptor, EPCR. Using Tie2-EPCR animals, where endothelial cells over-express EPCR and exhibit enhanced aPC-EPCR signaling, no significant reduction in cellular infiltration or fibrosis was evident with AngII infusion suggesting aPC-mediate protection is endothelial cell independent. Isolated infiltrating cells expressed significant EPCR transcripts suggesting a direct effect on infiltrating cells.

**Conclusions:**

This data indicates that aPC treatment abrogates the fibrogenic response to AngII. aPC does not appear to confer protection by stimulating the endothelium but by acting directly on the infiltrating cells, potentially inhibiting migration or activation.

## Background

Myocardial fibrosis is a common sequelae associated with many cardiovascular diseases and is characterized by the accumulation of excess extracellular (ECM) proteins within the myocardial tissues [1]. While initially the ECM deposition occurs in response to injury and is believed to be beneficial, it can become pathologic [1]. The lack of regression in this ECM deposition is the hallmark of myocardial fibrosis that ultimately can results in irreversible organ failure [1]. Current pharmacological agents used to treat cardiovascular disease do not abrogate or reverse myocardial fibrosis [2].

The etiology of myocardial fibrosis is multifactorial. It is associated with cellular and structural changes including the recruitment/proliferation of effector cells, apoptosis of myocytes and the accumulation and rearrangement of structural fibers [1], [3], [4]. Using the well-established AngII infusion model of myocardial fibrosis, we have previously investigated the early events in this process [3], [5]. Infusion of AngII in this model results in rapid infiltration of fibroblast progenitor cells, termed fibrocytes. Fibrocytes are capable of producing both pro-inflammatory mediators and fibrogenic factors[3], [5], [6]. They are believed to be capable of bridging an initial inflammatory response to a later corresponding fibrotic response [7], [8]. Fibrocyte infiltration is followed by a loss of functional myocytes and an accumulation of extracellular matrix proteins within the myocardial tissue resulting in the development of myocardial fibrosis [3], [4], [6].

In a mouse model of bleomycin induced lung fibrosis, where fibrocytes have been shown to be a key effector cell, administration of activated Protein C (aPC) decreased cellular infiltration and reduced tissue fibrosis [9], [10]. aPC is a serine protease that acts as a naturally produced anticoagulant by cleaving Factor Va and VIIIa, which inhibits thrombin formation [11]. Recently, aPC has been shown to have anti-inflammatory and cytoprotective activities as well [12], [13], [14]. The administration of aPC confers protection against sepsis and ischemia/reperfusion injury in kidney, skeletal muscle, skin grafts and myocardium [12], [14], [15], [16], [17], [18], [19]. These protective effects are believed to be due to the decreased vascular expression of cellular adhesion molecules; downregulation of pro-inflammatory mediator expression; the improvement of microvascular circulation and endothelial barrier function; and upregulation of survival signaling pathways [20], [21], [22], [23], [24]. aPC also appears to have direct beneficial effects on the myocardium such that individuals with a genetic defect within the aPC signaling pathway have an increased risk of cardiovascular events including myocardial infarcts[25], [26]. However, the benefits of aPC have yet to be tested in models of myocardial fibrosis in the absence of ischemia reperfusion.

These data suggested to us that aPC administration might modulate the myocardial response to elevated circulating AngII and potentially confer protection against the development of fibrosis secondary to AngII exposure.

## Materials and Methods

### Ethics

All work was performed in accordance with the Canadian Council on Animal Care and was approved by Dalhousie University’s University Committee on Laboratory Animals (REB #2007-1534). Animals were monitored for any signs of morbidity during the course of this study and all efforts were made to minimize suffering.

### Animals

Male C57BL/6 (Jackson Laboratory; Bar Harbour, ME) and Tie2-EPCR (generously provided by Dr. Charles Esmon, University of Oklahoma Health Science Centre, OK) mice ranging from 8–9 wk old. Mice were provided food and water *ad libitum* for at least 1 wk prior to experimentation. Animals were anesthetized with isoflurane (Baxter Healthcare Corp., New Providence, NJ) in oxygen delivered by a Fortec vaporizer. When surgical levels of anesthesia were reached a 1–2 cm mid-scapular skin incision was made and a mini osmotic pump (Alzet, Palo Alto, CA) was inserted subcutaneously. The incision was closed using 7 mm wound clips. Animals were randomly assigned to receive AngII (2.0 µg/kg/min, Sigma Aldrich, Oakville ON), aPC (0.4 µg/kg/min, Sigma Aldrich), AngII and aPC, AngII and Heparin (200 U/kg bolus injection at initiation and 70 U/kg/hr, MedXL, Montreal, QC) or a vehicle control of saline. The pumps remained in for 3d during which the animals were provided with food and water *ad libitum* and observed for signs of morbidity. Prior to euthanization, blood pressure measurements were taken via the Coda2 non-invasive cuff system (Kent Scientific, Torrington, CT) for a minimum of 5 consecutive measurements per animal. Animals were then anesthetized with isoflurane again and animals were sacrificed via exsanguination. Hearts were harvested and processed for cellular isolation or histologic and molecular analysis. Hearts intended for histologic and molecular analysis were divided along the short axis into 3 portions including the base, middle and apical sections. The base portion was processed for histological examination while the other two portions were snap frozen immediately for molecular analysis. Due to overlapping experimental groups previously published[3], some historical samples from AngII and saline infused animals were used for this study to compare to new experimental groups. Additional animals were added to saline and AngII experimental groups to ensure experimental reproducibility.

### Cellular Isolation and Culture

Infiltrating cells were isolated from excised heart of AngII infused animals as previously stated [3]. Cellular isolates were incubated at 37°C with 5% CO_2_ for 3d, after which all non-adherent cellular debris was removed and fresh media was supplied for an additional day. Cells were washed with sterile PBS and fixed for histologic staining or lysed using TRIzol (Gibco). Coverslips were stored at 4°C until stained and lysed samples were stored at −80°C until RNA isolation could be completed.

### Histological Analysis

Hearts were fixed in 10% formalin, paraffin embedded and serially sectioned (5 µm). Basic myocardial histology and cellular infiltration were examined using heart cross-sections stained with H&E. A blinded observer quantified infiltrating cells by counting mononuclear cells evident in between myocytes and around vessels in 5 fields of view at 25× magnification per animal.

### Collagen Deposition

Collagen detection was accomplished using Sirius red and fast green stains. Images of entire cross-sections of myocardium where compiled from images taken at 5× magnification. Using Adobe Photoshop CS5, red pixels were positively selected and summed for a total number of red (collagen) pixels. Subsequently, all non-background pixels were summed for a total number of heart pixels. The total collagen pixels were divided by the total heart pixels to provide a semi-quantitative measurement of the percent of the heart affected by fibrosis. To avoid measuring discrepancies between staining, the same red colour palette was used to select red pixels.

### TUNEL

Apoptosis was detected on 5 µm paraffin embedded heart sections using a commercially available terminal deoxynucleotide transferase-mediated dUTP nick-end labeling (TUNEL) assay (Chemicon, Billerica, MA). Assay was completed as per manufacturers instructions. Five images from a heart section from each animal were viewed with the 25× objective. An observer blinded to the treatment group counted the number of TUNEL positive cells in each image, and the mean number of cells per image for each experimental group was compared to the control group.

### Immunofluorescence Staining

Isolated cells were grown on coverslips and then fixed in 4% paraformaldehyde, permeabilized with 0.03% Triton X-100, blocked against non-specific antibody binding with 10% normal goat serum and stained for CD45 (BD Biosciences, Mississauga, ON) and Collagen type 1 (Rockland Inc, Gilbertsville, PA). Antibodies were detected using anti-host specific Alexa fluorescently labeled secondary antibodies (Invitrogen).

### RNA Isolation and cDNA Synthesis

Using the TRIzol reagent (Gibco), RNA was isolated from the snap frozen myocardium as per the manufacturer’s protocol. First strand cDNA was synthesized from 1 µg of total RNA using iScript cDNA Synthesis Kit (Biorad. Hercules, CA).

### Conventional Reverse Transcription-Polymerase Chain Reaction (RT- PCR)

Conventional RT-PCR was completed using 12.5 ng of input cDNA with 0.5 µM of each of the forward and reverse primers and 10 uL of REDExtract-N-Amp PCR Ready Mix (Sigma) that was subjected to conventional RT-PCR using an iCycler (BioRad). Experimental samples were run along with a sample containing no template cDNA to ensure reagents were contamination free. All samples were run on a 1% agarose gel and were visualized using ethidium bromide and UV imaging conducted using an AlphaImager (Alpha Innotech, San Leanardo, CA). An image of PCR products was produced for analysis. The primers were designed against the mRNA sequence of endothelial protein C receptor (EPCR) (forward: 5′-GCCCTTTGTAACTCCGATGG-3′; reverse: 5′-GGAGGATGGTGACGTTTTGG-3′); and 18S ribosomal RNA (forward: 5′-TCAACTTTCGATGGTAGTCGCCGT-3′; reverse: 5′-TCCTTGGATGTGGTAGCCGTTTCT-3′).

### Relative Quantitative RT-PCR

Relative quantitative RT-PCR (qRT-PCR) was completed using 12.5 ng of input cDNA with 0.5 µM of each of the forward and reverse primers and 1x iQ SYBR Green Supermix (Bio-Rad) was subjected to qRT-PCR using an iQ Multicolour Real-Time PCR Detection System thermocycler (Bio-Rad). Standard curves, for efficiency, and no-template control samples were run along with the samples during thermocycling. A melting curve was performed after thermocycling was complete to ensure target specificity. The primers were designed against the mRNA sequence of connective tissue growth factor (CTGF) (forward: 5′-TCAACCTCAGACACTGGTTTCG-3′; reverse: 5′-TAGAGCAGGTCTGTCTGCAAGC-3′); transforming growth factor β1 (TGF-β1) (forward: 5′-GGTCTCCCAAGGAAAGGTAGG-3′; reverse: 5′-CTCTTGAGTCCCTCGCATCC-3′), platelet derived growth factor (PDGF) (forward: 5′-TTAAGGACTTGACCCTGCTTCC-3′; reverse: 5′-GCATCTGCCTGAAGTGTGTACC-3′); collagen 1α1 (forward: 5′-CAACAGTCGCTTCACCTACAGC-3′; reverse: 5′-GTGGAGGGAGTTTACACGAAGC-3′); B-cell lymphoma 2 (Bcl-2) (forward: CCAGCGTGTGTGTGCAAGTGTAAA-3′; reverse: 5′-ACACTCCGGCTTCACTGAGAATGT-3′); Bcl-2 associated X protein (Bax) (forward: 5′-tggttgccctcttctactttgc-3′; reverse: 5′-CATCTTCTTCCAGATGGTGAGC-3′); and 18S, as stated above. Expression was normalized to the 18S ribosomal gene using the Pfaffl method.

### Statistical Analysis

One-way ANOVA tests were completed on all quantitative data using the Bonferonni post-test to compare all the experimental groups in multi-treatment experiments. A students T-test was used to compare an experimental group to a control group if there was only one experimental group. Our level of significance was set as p≤0.05. All statistical calculations were computed using GraphPad Prism 4 software.

## Results

### Hemodynamic Measurements

**Figure 1:**
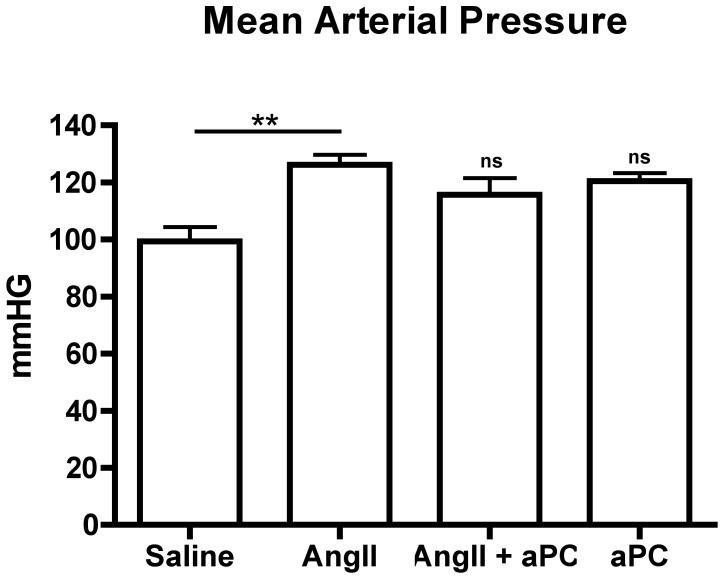
Hemodynamic Measurements. AngII infusion after 3d was associated with a significant increase in the mean arterial pressure (mmHG) of mice as measured by a non-invasive tail cuff system. Co-infusion with aPC appears to modestly inhibit AngII induced hypertension. *p<0.05 **p<0.01.

To assess the impact of aPC treatment in a model of myocardial fibrosis secondary to AngII infusion, we assigned animals to four experimental groups. Animals were infused with: a) AngII (2.0 µg/kg/min, n = 11); b) AngII with aPC (0.4 µg/kg/min; n = 6); aPC alone; or c) saline (control, n = 12). Infusion of AngII, a known vasoconstrictor, resulted in a significant increase in mean arterial blood pressure when compared to saline control (126.2±3.5 mmHg and 99.3±5.1 mmHg respectively; p<0.01; Fig. 1). The addition of aPC failed to reduce the blood pressure elevation caused by AngII back to baseline levels evident in saline controls.

### Cellular Infiltration

**Figure 2:**
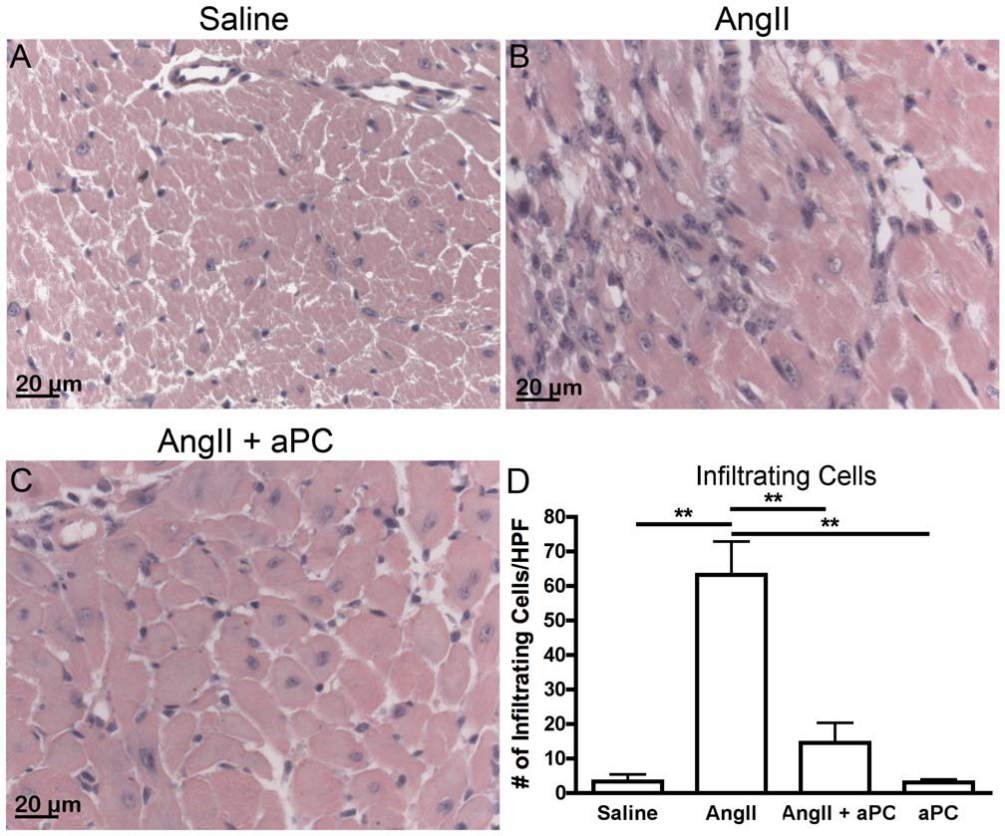
Cellular Infiltration. Representative images of hearts sections stained with H&E from animal infused with saline (A), AngII (B) and AngII and aPC (C). AngII infusion was associated with cellular infiltration within the interstitial and perivascular tissues. Evident cellular infiltration can be observed as non-myocyte cells that are mononuclear with little cytoplasm and are typically found within focal clusters (example in panel B on the left side of the micrograph). Little cellular infiltration was evident when aPC was also infused. Infiltrating cell counts were completed to quantify this observation within an average of 5 field of views/heart (D). Significant infiltration was measured in AngII infused animals compared to saline controls but no significant increase in cell infiltration could be detected between aPC or AngII-aPC and saline infused animals. Images were taken at 40× magnification. *p<0.05.

Myocardial fibrosis is associated with a significant cellular component which we assessed using H&E stained myocardial cross-sections from each of our experimental groups. While normal myocardial histology was observed in saline controls, cellular infiltration was evident within the myocardial tissue from AngII infused animals (Fig. 2A, B). Little cellular infiltration was found in the myocardium of AngII-aPC infused animals, which appeared similar to saline controls (Fig. 2C). This observation was confirmed by quantification of cellular infiltration that indicated significant cellular infiltration in AngII infused animals compared to controls (63.1±9.7 cells/field of view (FOV) and 3.3±1.9 cells/FOV; p<0.05; Fig. 2D). No significant cellular infiltration was detected in AngII-aPC treated animals or when aPC was infused alone compared to saline control (14.5±5.8 cells/FOV and 3.0±0.8 cells/FOV; Fig. 2D).

### Myocardial Fibrosis

**Figure 3:**
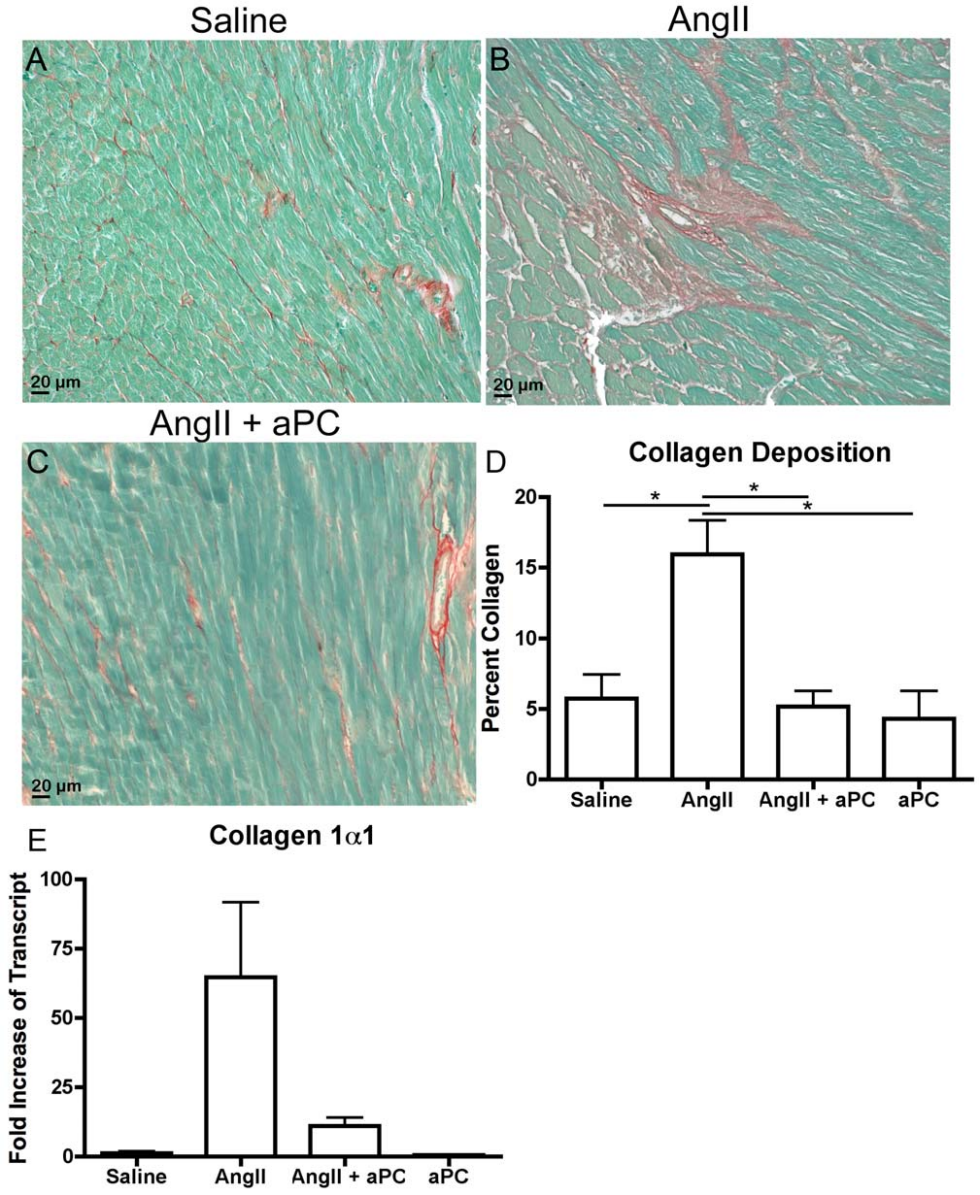
Collagen Deposition. Myocardial collagen content was assessed as an indicator of fibrosis. Collagen was initially assessed using myocardial sections stained with Sirius red and fast green where collagen is stained red and the counter stain is green. Representative images of sections from animals infused with saline (A), AngII (B) and AngII and aPC (C). Collagen content of Sirius red stained sections was semi-quantitatively assessed by completing red pixel measurements compared to total pixel content to derive a percent collagen content within a cross section of heart (D). qRT-PCR was completed to assess pro-collagen1α1 transcript levels within the different experimental groups (E). Images were taken at 25× magnification **p<0.01.

To assess whether the cellular infiltration was accompanied by fibrosis, the degree of myocardial fibrosis in experimental animals was determined by staining myocardial cross-sections with Sirius red. Excess collagen (red) deposition was evident within multi-focal areas of the myocardium from AngII infused animals (Fig. 3A,B). However, Collagen levels in AngII-aPC infused animals appeared similar to saline controls (Fig. 3C). Quantification of collagen validated our observation of significant collagen deposition in AngII infused animals, which was significantly inhibited by the coadministration of aPC (saline: 5.7±1.7%; AngII:16.5±2.2%; AngII-aPC: 5.2±1.1%; p<0.01, Fig. 3D). Infusion of aPC by itself resulted in baseline levels of collagen deposition (4.3±2.0%). Transcript levels of pro-collagen1α1, assessed by qRT-PCR, appeared decreased in AngII-aPC infused animals compared to animals infused with AngII alone, however, statistical significance was not reached (Fig. 3E). Taken together, aPC treatment inhibited AngII-induced collagen production and deposition.

### Pro-fibrotic Factors

**Figure 4:**
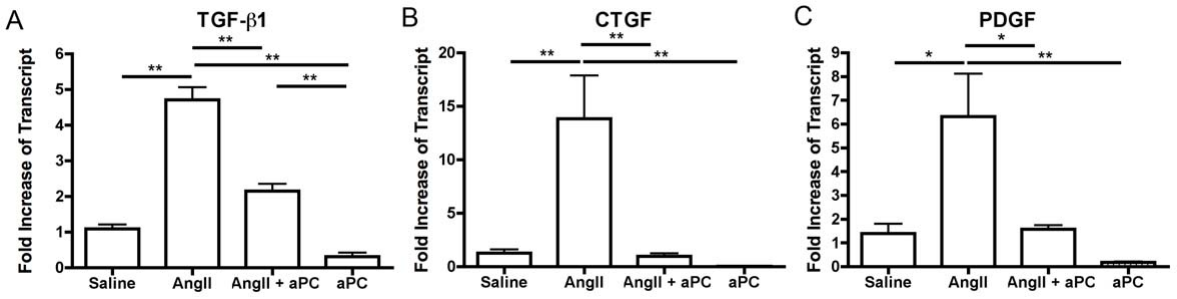
Pro-fibrotic Mediators. The transcript levels of different pro-fibrotic mediators were assessed using qRT-PCR from whole heart samples. TGF-β1, CTGF and PDGF transcript levels were significantly elevated in animals exposed to only AngII but transcript levels were significantly less when aPC was also infused. *p<0.05 **p<0.01.

The transcript levels of well-known fibrotic factors were assessed by qRT-PCR to determine if their expression levels were altered by aPC treatment in this fibrotic model. We assessed TGF-β1, CTGF and PDGF transcript levels because they have previously been shown to be increased in response to AngII infusion and are believed to promote the initiation and progression of fibrosis [3], [27]. All three pro-fibrotic factors where significantly increased in AngII infused animals (TGF-β1: 4.7±0.3 fold; CTGF: 13.8±4.0 fold; PDGF: 6.3±1.8 fold) but not when aPC was infused with AngII (Fig. 4). In fact, both CTGF and PDGF levels were measured at control levels when aPC was co-infused. Furthermore, aPC infusion alone appeared to decrease the transcript levels of all three fibrotic factors below baseline levels, though significance was not achieved (Fig. 4).

### The Effect of aPC on the Endothelium

**Figure 5:**
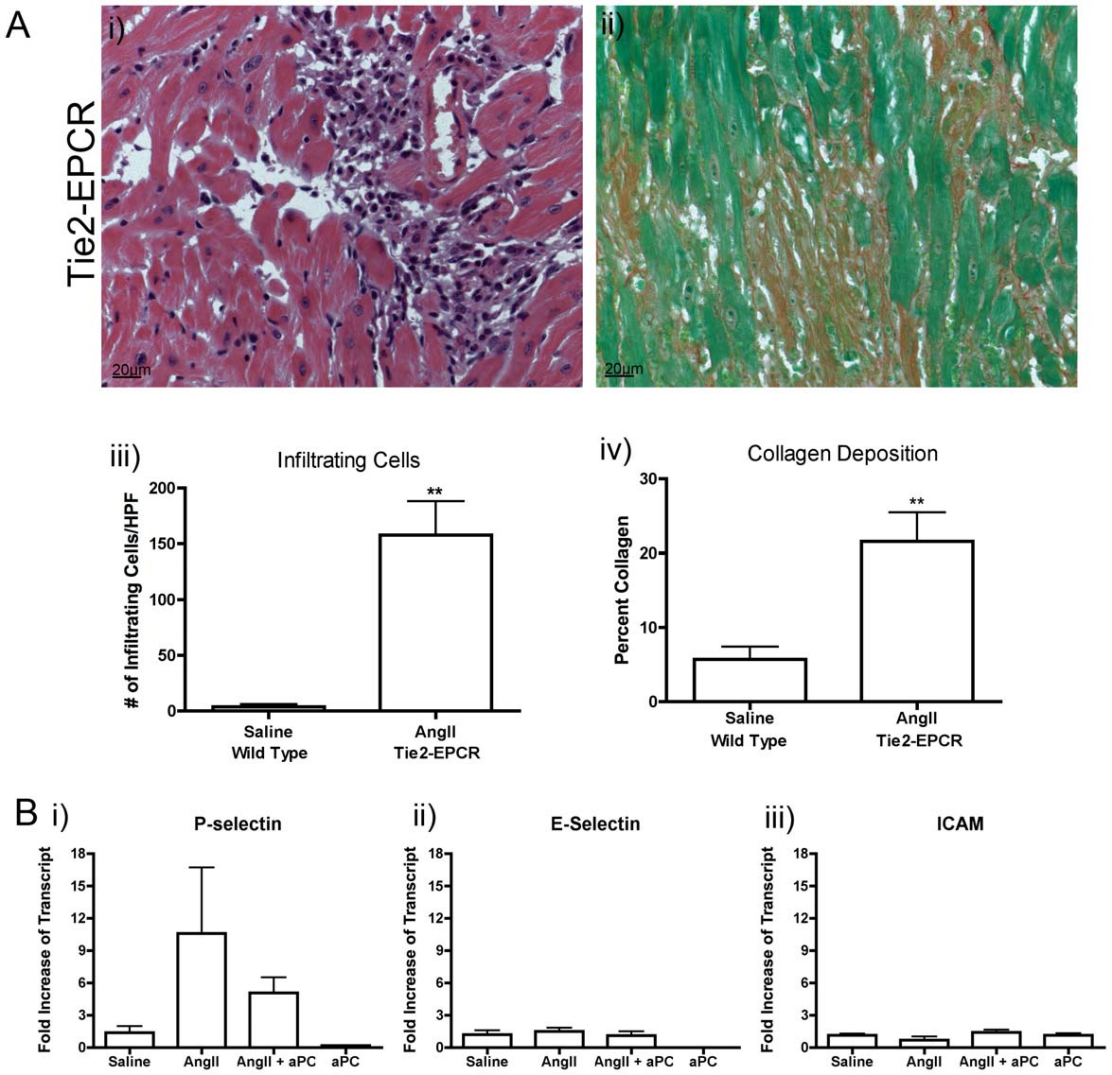
Endothelial Cell Activation. AngII was infused in Tie2-EPCR mice that have excess expression of the aPC receptor within endothelial cells and as such have chronically downregulated endothelial cell activation (A). Cellular infiltration and collagen deposition, as measured by H&E (Ai) and Sirius red (Aii) staining respectively, were still evident within AngII infused Tie2-EPCR animals. Quantification of both cellular infiltration and collagen content demonstrated a significant increase beyond the levels of saline controls in wild type animals (Aiii, Aiv). The transcript levels of cellular adhesion molecules, including P-selectin (Bi), E-selectin (Bii) and ICAM (Biii), were assessed using qRT-PCR analysis of whole heart samples. No significant change was measured in the level of cellular adhesion molecule transcript levels in either experimental group compared to saline controls (B). Images were taken at 40× magnification. *p<0.05 **p<0.01.

aPC is believed to mediate many of its anti-inflammatory and cytoprotective effects by engagement of EPCR on the endothelium, which inhibits endothelial cell activation and promotes endothelial barrier function [20], [21], [28]. To evaluate the role of the endothelium in aPC-mediated inhibition of AngII induced myocardial fibrosis, Tie2-EPCR transgenic mice that overexpress EPCR on endothelial cells, were used. These animals have been previously shown to have decreased expression of cellular adhesion molecules on the endothelium, indicative of chronic endothelial aPC-EPCR signaling [24], [29]. These mice have been shown to be significantly resistance to endotoxin-induced death as well as cancer metastasis [24], [29]. Interestingly, AngII-treated Tie2-EPCR mice developed significant myocardial cellular infiltration as well as collagen deposition when compared to wild type, saline treated animals (Fig. 5A). Taken together, this data suggests that aPC does not appear to elicit its protective effects on the myocardium (fibrosis reduction) via activation of the EPCR on the endothelium.

We also investigated the transcript levels of P-selectin, E-selectin and ICAM-1 in the experimental wild-type groups to determine if there was a detectable alteration in gene expression between groups. We measured an apparent increase in P-selectin transcripts in AngII exposed hearts in comparison to saline controls and AngII-aPC exposed animals, though it did not achieve statistical significance (Fig. 5B). There was no detectable difference in the transcript levels of E-selectin or ICAM-1 in any of the experimental groups (Fig. 5B). aPC infusion appeared to reduce the expression of P-selectin and E-selectin below levels evident in saline controls, though the difference was not found to be statistically significant (Fig. 5B). These findings support of our observation in Tie2-EPCR mice as these adhesion molecules are normally expressed on endothelial cells and are used as markers of activation.

### Apoptosis

**Figure 6:**
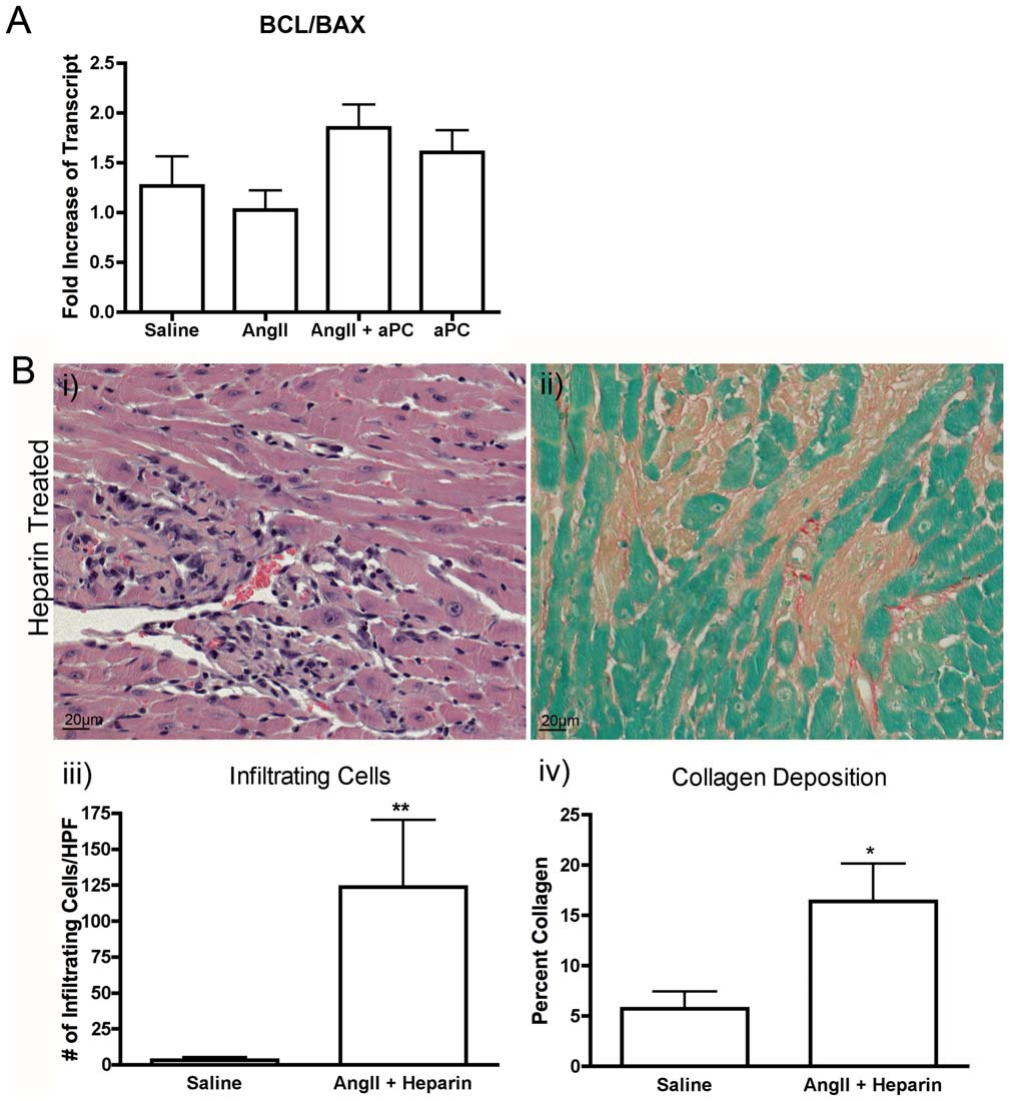
Putative Mechanism of Action of aPC. Transcript levels of the pro-survival factor Bcl-2 and the pro-apoptotic factor Bax were measured by qRT-PCR and a ratio of the two genes were generated for each animal assessed from each experimental group (A). No significant difference in the Bcl-2/Bax ratio was measured. To assess the effect of anticoagulation in this model, animals were infused with both AngII and heparin to determine the effects of anticoagulation within our fibrotic model (B). Cellular infiltration and collagen deposition, as measured by H&E (Bi) and Sirius red (Bii) staining respectively, were still evident within the myocardium of animals infused with AngII and heparin. Quantification of both cellular infiltration (Biii) and collagen content (Biv) demonstrated a significant increase beyond the levels of saline controls. Images were taken at 40× magnification. **p<0.01.

aPC is known to have cytoprotective properties that arise from its inhibition of apoptotic signaling pathways [22]. We quantified the extent of cell death by apoptosis between experimental groups using a TUNEL stain. We did not find a significant number of TUNEL positive cells within the myocardium of AngII infused animals when compared to saline control (data not shown). We then assessed the transcript levels of the pro-survival molecule, Bcl-2, and the pro-apoptosis molecule, Bax, to determine if the ratio of these two apoptosis regulators deviated from that of our saline controls. We found no significant differences in the Bcl-2/Bax ratio in any of our experimental groups (Fig. 6A). This data suggests that apoptosis is not a significant contributor to the fibrotic process at this time point. Therefore, the cytoprotective effects of aPC would not be a necessary mechanism to confer protection against the fibrotic process evident in this model.

### Effects of Anticoagulation on Fibrosis Development

aPC is best known for its role in the coagulation pathway where it inhibits the production of thrombin by cleaving upstream mediators required for its generation, promoting anticoagulation [11]. To determine if the beneficial effect of aPC on fibrosis development in our experiments resulted from anticoagulant activities we co-infused AngII with heparin (n = 3), an anticoagulant which also reduces thrombin levels[30]. In animals infused with AngII and heparin, significant cellular infiltration and fibrosis were still evident suggesting that the anticoagulant activities of aPC are likely not responsible for inhibiting the development of myocardial fibrosis (Fig. 6B).

### EPCR Expression on Infiltrating Cells

**Figure 7:**
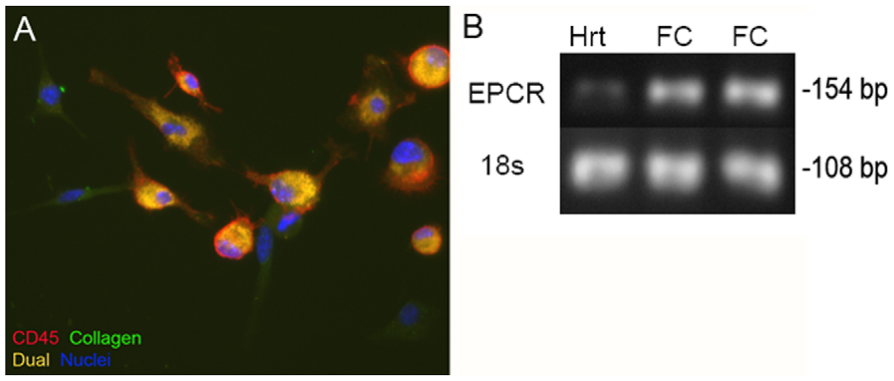
EPCR Expression on Infiltrating Cells. Infiltrating cells were isolated from heart exposed to AngII for 3d and placed in culture for 3d (A). The phenotype of cultured cells were determined by staining for CD45 (red), Collagen (green) and nuclei (blue). The overlay appears yellow. Conventional RT-PCR was used on samples isolated from these cultured cells to analyze the expression of EPCR in fibrocytes rich cultures (FC, n = 2 - cultured cells from 2 different animals) isolated from AngII infused hearts (B). Significant EPCR expression was detected in isolated cells as well as faint expression in a sample of whole heart (HRT). 18s was used as a housekeeping gene to ensure equal loading between samples.

EPCR expression has been found on several subsets of circulating leukocytes and as such, these cells are also capable of responding to elevated circulating aPC [31], [32]. With a lack of benefit seen with AngII infusion in Tie2-EPCR mice, we don’t believe the endothelial cells are the main cell type conferring protection against AngII-mediated fibrosis. As such, we wanted to investigate if fibrocytes, a circulating leukocyte subset, express EPCR and are capable of responding to aPC stimulation. Infiltrating fibrocytes were isolated out of the hearts of 3d AngII infused animals as previously described [3]. After 3 days in culture, immunofluorescence for CD45 and collagen1 expression was used to elucidate their phenotype. Approximately 50% of isolated cells were CD45^+^/Collagen^+^, indicative of a fibrocyte rich population (Fig. 7A). We also assessed the expression level of EPCR on isolated infiltrating cells via conventional RT-PCR to determine if they may directly respond to increased circulating aPC levels. We found substantial levels of EPCR transcripts expressed by isolated cells (FC) as well as the control native myocardium (HRT; Fig. 7B). This data confirms that these cells do express EPCR and could be directly affected by increased levels of circulating aPC.

## Discussion

Initially known as a vasoconstrictor, AngII has more recently been shown to be a pro-fibrotic molecule. It is involved in the development of myocardial fibrosis both clinically and in animal models [1], [3], [6]. AngII infusion in mice has therefore become a well-established model used to study myocardial fibrosis. Using this model we have previously demonstrated that AngII results in significant early cellular infiltration (1d) by fibroblasts progenitor cells (fibrocytes) followed by corresponding deposition of extracellular matrix proteins by 3d [3], [5]. We and others have therefore been able to use this model of fibrosis to study early mediators of the fibrotic process in an effort to identify potential interventions.

In this study we demonstrate that aPC protects against the development of myocardial fibrosis secondary to AngII exposure. We provide novel evidence that aPC inhibits the previously described fibrocyte accumulation followed by collagen deposition seen with AngII infusion. Furthermore, we show a corresponding inhibition of expression of pro-fibrotic mediators, specifically TGF-β1, CTGF and PDGF, with aPC treatment. Our findings support previous work demonstrating that aPC administration during a myocardial ischemic insult results in reduced scarring and improved function, though the mechanisms by which this occurs have not been elucidated [18], [23].

We have previously established that fibrosis is temporally preceded by myocardial infiltration of fibrocytes, that are likely responsible for the development of fibrosis[3]. Work by others [6], [33] has demonstrated a link between fibrocyte accumulation and fibrosis by using strategies that reduced fibrocyte migration and consequently decreased fibrosis. In our model of myocardial fibrosis, administration of aPC blocked fibrocyte infiltration normally associated with AngII exposure. This inhibition in cellular recruitment does not appear to be in response to decreased expression of cellular adhesion molecules normally expressed on endothelial cells. We were unable to show any significant differences in mRNA expression levels for P-selectin, E-selectin or ICAM in heart homogenates between saline, AngII or AngII-aPC infused animals. While aPC infusion alone appeared to decrease the levels of P-selectin and E-selectin below baseline levels, suggesting that it does directly inhibit the expression of individual cellular adhesion molecules, these differences did not reach statistical significance. It is possible that an increase in transcript levels of various cellular adhesion molecules may have been masked by a dilution of their specific transcript levels in the endothelium by the overwhelming amount of transcripts from other cell types within the myocardium.

We sought to isolate the effects of aPC on the endothelium, as endothelial cells represent the point of entry for circulating fibrocytes into the myocardium. We used Tie2-EPCR mice, which over express the EPCR on the endothelium, creating an animal in which there is increased endogenous aPC-EPCR signaling. However, Tie2-EPCR mice that received exogenous AngII still developed significant cellular infiltration and corresponding fibrosis within the myocardium. Taken together, our data suggests that the mechanisms responsible for the anti-fibrotic effects of aPCs do not appear to be mediated through EPCR signaling on endothelium.

A mechanism by which aPC might be cardioprotecitve may relate to its cytoprotective and anti-apoptotic signaling capabilities. In the present study we were unable to demonstrate significant apoptosis within the myocardium of AngII infused animals by TUNEL staining and we could not show a difference in the ratio of pro-survival and pro-apoptosis mediators, Bcl-2/Bax, in animals that received AngII+aPC. While this data does not rule out a cytoprotective effect of aPC within this model at a different time point, it did not appear to be the key mechanism by which aPC confers its initial anti-fibrotic effects.

Though there is little evidence that the protective effects of aPC are mediated by its anticoagulative properties, there is some evidence suggesting that anticoagulation can be protective in hypertension [34], [35]. To address this limitation we infused mice with AngII along with an alternative anticoagulant, heparin. We found that AngII infused mice that also received heparin showed similar cellular infiltration and fibrotic sequelae as those that receive AngII alone. Our findings indicate that the anticoagulant properties of aPC are an unlikely mechanism providing its protection effects.

Though this study shows that aPC administration is effective in counteracting the fibrotic effects that occur secondary to AngII infusion, it does not appear to be mediated by the classic signaling effects of systemic aPC administration. Our findings suggest that aPC had no detectable effect on endothelial cells, did not increase cytoprotective signaling and that its effects were not dependent on its anticoagulation properties. The beneficial effects of aPC seemed to be mediated by a reduction in cellular infiltration and a reduction in myocardial pro-fibrotic mediator production (CTGF, PDGF and TGF-β1). It is likely that aPC is mediating these effects by directly stimulating a cell type other than endothelial cells that is involved in the development of myocardial fibrosis. EPCR expression has been found in monocytes, the lineage from which fibrocytes originate, and myofibroblasts, the phenotype which fibrocytes differentiate into [36], [37]. We detected EPCR expression in fibrocyte rich cultures isolated from the myocardium suggesting that fibrocytes themselves express EPCR. This would enable fibrocytes to directly respond to aPC. Interestingly, aPC has been shown to directly inhibit monocyte activation and migration in the presence of potent stimulators, including TNFα and CCL2, respectively [31], [38]. It is therefore possible that systemic administration of aPC may have a direct and similar effect on circulating fibrocytes and may inhibit their activation and recruitment into the myocardium. This potential mechanism intuitively explains the decreased cellular infiltration within the heart. Furthermore, fibrocytes are known to produce pro-fibrotic mediators when recruited into tissue and if aPC does inhibits their recruitment and/or activation, this would address the significant reduction of the pro-fibrotic factors in the myocardium seen when aPC is added to our model of myocardial fibrosis. Further work is required to understand the effect of aPC on fibrocytes and the effect of aPC on the heart in the presence of fibrotic stimuli.

Myocardial fibrosis is part of the continuum that eventually contributes to the development of chronic heart failure [1]. Even with current medical interventions, the prognosis for patients with heart failure is not always promising, forcing researchers to consider new therapies [39]. Knowledge gained from the present study on the beneficial effects of aPC may offer some insight into novel approaches to reduce the burden of cardiovascular diseases.
